# Supplementary material for: Preterm Infants on Early Solid Foods and Iron Status in the First Year of Life—A Secondary Outcome Analysis of a Randomized Controlled Trial
Source: Nutrients. 2022 Jun 30;14(13):2732. doi: 10.3390/nu14132732 (PMC9269052; doi:10.3390/nu14132732)
Supplement: Supplementary file 1 [file nutrients-14-02732-s001.zip › nutrients-1795567-supplementary/Supplementalmaterial/PIES_Iron_tableS1-milk_2022-06-14.pdf]

Table S1. Iron status in infants according to feeding type at discharge

Parameter	*6 weeks corrected age*		*6 months corrected age*		*12 months corrected age*	
	**Breastfed** (n=55)	**Formula** (n=111)	**Breastfed** (n=55)	**Formula** (n=111)	**Breastfed** (n=55)	**Formula** (n=111)
**Ferritin (mcg/L)**	**46.1 (34)**	**48.7 (43.5)**	**28.5 (20)**	**34.1 (24)**	**28.5 (15)**	**28.7 (21.6)**
Hemoglobin (g/dL)	10.7 (1.2)	11.2 (1.1)*	12.1 (0.8)	12.3 (0.8)	12.2 (0.7)	12.4 (0.9)
Hematocrit (%)	31.1 (3.3)	32.1 (3.1)*	34.4 (2.1)	34.8 (2.2)	34.6 (3.8)	35.5 (2.7)
MCV (fl)	77.1 (3.5)	79 (3.7)*	74 (3.5)	74.5 (3.1)	74.6 (3.5)	74.9 (3.6)
Transferrin (g/L)	2.3 (0.4)	2.5 (0.3)	2.6 (0.3)	2.6 (0.3)	2.7 (0.3)	2.8 (0.4)
sTFR (mg/L)	2.7 (4.9)	9.3 (68.8)	1.8 (0.7)	1.6 (0.4)	1.7 (0.3)	1.7 (0.3)
Iron (mcg/dL)	88.6 (38.5)	85.3 (27.8)	66.4 (21.5)	71.8 (22.9)	61.5 (24.8)	63.6 (25)
Transferrin saturation (%)	27.5 (12.5)	24.5 (8.9)	18.2 (6.1)	19.6 (7.3)	16.1 (6.5)	16.4 (7.3)
Iron deficiency and anemia						
Iron deficiency	25 (45.5)	57 (51.4)	6 (10.9)	3 (2.7)*	1 (1.8)	7 (6.3)
Anemia	2 (3.6)	0 (0)	0 (0)	0 (0)	0 (0)	0 (0)
Iron deficiency anemia	3 (5.5)	0 (0)*	0 (0)	3 (2.7)	0 (0)	0 (0)

Data are presented as mean and standard deviation in parentheses. Iron deficiency, anemia, and iron deficiency anemia are presented as number of patients and percentage in parentheses. P values <.05 were considered statistically significant, parameters with significant differences before correction for multiple testing were marked with *. After correction for multiple testing (Bonferroni), no significant differences were detected.
