# Supplementary material for: Preterm Infants on Early Solid Foods and Iron Status in the First Year of Life—A Secondary Outcome Analysis of a Randomized Controlled Trial
Source: Nutrients. 2022 Jun 30;14(13):2732. doi: 10.3390/nu14132732 (PMC9269052; doi:10.3390/nu14132732)
Supplement: Supplementary file 1 [file nutrients-14-02732-s001.zip › nutrients-1795567-supplementary/Supplementalmaterial/PIES_Iron_tableS2-SGA_2022-06-14.pdf]

Table S2. Iron status in infants born small for gestational age

Parameter	*6 weeks corrected age*		*6 months corrected age*		*12 months corrected age*	
	**SGA** (n=13)	**AGA** (n=164)	**SGA** (n=13)	**AGA** (n=164)	**SGA** (n=13)	**AGA** (n=164)
**Ferritin (mcg/L)**	**58.4 (34)**	**45.3 (39.8)**	**37.6 (32.6)**	**31.8 (21.8)**	**34 (45.2)**	**30.9 (27.7)**
Hemoglobin (g/dL)	10.5 (0.8)	11.1 (1.1)	11.8 (0.6)	12.3 (0.8)*	12.2 (0.8)	12.3 (0.8)
Hematocrit (%)	30.6 (1.8)	31.9 (3.2)	33.9 (1.1)	34.8 (2.2)	35.1 (1.9)	35.1 (3.2)
MCV (fl)	78.4 (3.6)	78.4 (3.8)	74.2 (3.2)	74.4 (3.4)	75.7 (3.8)	74.6 (3.6)
Transferrin (g/L)	2.4 (0.3)	2.5 (0.4)	2.6 (0.4)	2.6 (0.3)	2.9 (0.6)	2.8 (0.3)
sTFR (mg/L)	1.5 (0.3)	7 (55)	1.6 (0.5)	10.3 (90.7)	1.6 (0.2)	1.7 (0.3)
Iron (mcg/dL)	93.8 (34.7)	86 (32.9)	74.4 (27.5)	70.3 (22.8)	63.4 (24.4)	62.5 (25.7)
Transferrin saturation (%)	27.8 (11.1)	25.3 (10.6)	19.7 (7.8)	19.4 (7.1)	16.8 (7.7)	16.1 (7)
Iron deficiency and anemia						
Iron deficiency	4 (30.8)	86 (52.4)	0 (0)	9 (5.5)	0 (0)	9 (5.5)
Anemia	0 (0)	2 (1.2)	0 (0)	0 (0)	0 (0)	0 (0)
Iron deficiency anemia	1 (7.7)	2 (1.2)	0 (0)	3 (1.8)	0 (0)	0 (0)

Data are presented as mean and standard deviation in parentheses. Iron deficiency, anemia, and iron deficiency anemia are presented as number of patients and percentage in parentheses. P values <.05 were considered statistically significant, parameters with significant differences before correction for multiple testing were marked with *. After correction for multiple testing (Bonferroni), no significant differences were detected.

SGA – small for gestational age (<10. percentile), AGA – appropriate for gestational age
