# Supplementary material for: Preterm Infants on Early Solid Foods and Iron Status in the First Year of Life—A Secondary Outcome Analysis of a Randomized Controlled Trial
Source: Nutrients. 2022 Jun 30;14(13):2732. doi: 10.3390/nu14132732 (PMC9269052; doi:10.3390/nu14132732)
Supplement: Supplementary file 1 [file nutrients-14-02732-s001.zip › nutrients-1795567-supplementary/Supplementalmaterial/PIES_Iron_tableS3-neuro_2022-06-14.pdf]

Table S3. Bayley-III at 12 months corrected age in infants with and without iron deficiency at 6 weeks and 6 months corrected age

**Bayley-III at 12 months corrected age**	**Infants without/with iron deficiency at 6 weeks corrected age**			**Infants without/with iron deficiency at 6 months corrected age**		
	**No iron deficiency** (n=78)	**Iron deficiency** (n=56)	**p-value**	**No iron deficiency** (n=117)	**Iron deficiency** (n=8)	**p-value**
Cognitive DE	88.3 (83.6-93.1)	92.3 (88.3-96.4)	.16	90.1 (86.5-93.8)	97.0 (84.4-109.6)	.26
Cognitive US	94.4 (90.2-98.5)	99.4 (95.9-102.9)	.06	97.5 (94.4-101.0)	102.5 (91.8-113.0)	.33
Motor DE	87.5 (82.5-92.5)	96.9 (92.6-101.1)	*.005*	92.7 (89.1-96.3)	104.6 (92.1-117.0)	.05
Motor US	85.5 (81.6-89.4)	92.9 (89.6-96.2)	*.005*	89.9 (87.2-92.7)	99.0 (89.4-108.6)	.05
Language DE	87.6 (83.0-92.2)	96.0 (92.2-99.9)	*.007*	91.9 (88.4-95.4)	101.1 (89.0-113.2)	.12
Language US	87.8 (84.4-91.2)	94.7 (91.8-97.5)	*.003*	91.7 (89.1-94.3)	96.9 (88.0-105.9)	.23

Data are presented as estimated marginal means and 95%CI, linear mixed models through sex and gestational age in days with a random intercept accounting for same family siblings. P values <.05 were considered statistically significant. After correction for multiple testing (Bonferroni), no significant differences were detected.
